# Recycling of Polyurethane Foams via Glycolysis: A Review

**DOI:** 10.3390/ma17184617

**Published:** 2024-09-20

**Authors:** Kinga Wieczorek, Przemysław Bukowski, Krystian Stawiński, Iwona Ryłko

**Affiliations:** 1 Institute of Agricultural Engineering, Wrocław University of Environmental and Life Sciences, 37 Chełmońskiego Str., 51-630 Wrocław, Poland; przemyslaw.bukowski@upwr.edu.pl (P.B.); iwona.rylko-polak@upwr.edu.pl (I.R.); 2 Selena Industrial Technologies Sp. z o.o., Pieszycka 3, 58-200 Dzierżoniów, Poland; krystian.stawinski@selena.com

**Keywords:** chemical recycling, polyurethane, glycolysis, closed-loop economy, rigid foam, flexible foam

## Abstract

Polyurethane foams constitute highly problematic waste due to their low density and consequently large volume. Among the most promising recycling approaches, the glycolysis of polyurethane waste stands out and was extensively discussed in this article. Existing literature reviews lack a detailed analysis of glycolysis processes and a clear presentation of the most important data. However, in this review, the scientific literature on glycolysis has been thoroughly examined and updated with the latest research in the field. The article provides an overview of glycolysis methods, categorized into rigid and flexible foams, along with a review of the catalysts and process conditions employed. Additionally, this study offers a comprehensive analysis of industrial methods protected by active patents, which has not been previously explored in the literature. This detailed examination of patent information adds significant value to the review and distinguishes it from others. Furthermore, this review also aims to introduce the main types of polyurethanes and their properties. It outlines the fundamentals of recycling strategies, thermomodernization trends, and environmental considerations, highlighting the critical role of recycling in the industry. The article serves as a complete foundation for exploring new alternative methods in this field.

## 1. Introduction

Polyurethane (PU) materials are common substances characterized by a broad spectrum of applications. It all began relatively recently; the first fundamental reaction of addition polymerization between diisocyanates and polyols was conducted in 1937. It was then that Otto Bayer and his research team synthesized the first polyurethane polymer in the laboratories of the German company IG Farben Industrie, in response to the work of Carothers on polyamides or nylons developed at E.I. du Pont [1]. However, the first foamed materials were not achieved until a decade after the aforementioned reaction was discovered. Flexible polymers, on the other hand, were synthesized by Hochtelen in 1952. These materials were swiftly acknowledged by the populace, prompting large-scale production in subsequent years. A pivotal advancement that notably expedited the progress in this domain was the formulation of a method for manufacturing foamed materials from polyether polyols, which substantially displaced the previously employed polyesters owing to their lower production costs [2]. So, over nearly 80 years, polyurethanes have become one of the most dynamically developing groups of polymers. This is all due to their segmented structure, as polyurethanes consist of both flexible and rigid segments [3]. By controlling several variables such as chemical composition, functionality, and molecular weight of individual reagents, a wide range of materials with distinctly different properties can be obtained [4]. Additionally, processing conditions and apparent density can be manipulated. They have successfully found applications as foams, which constitute over half of the total production of polymer foams [5].

**Figure 1:**
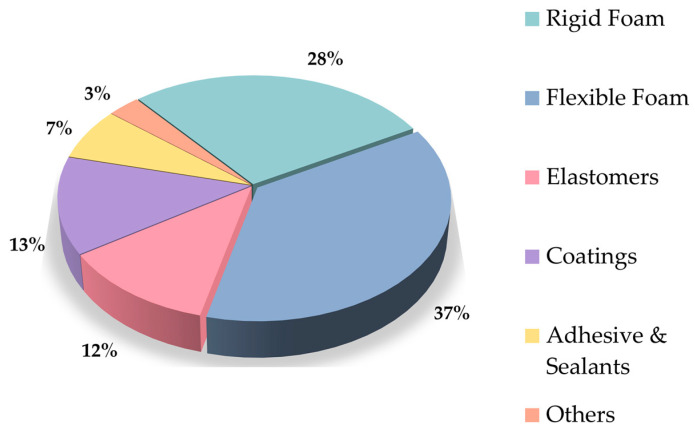
Global polyurethane market by applications [9].

**Figure 2:**
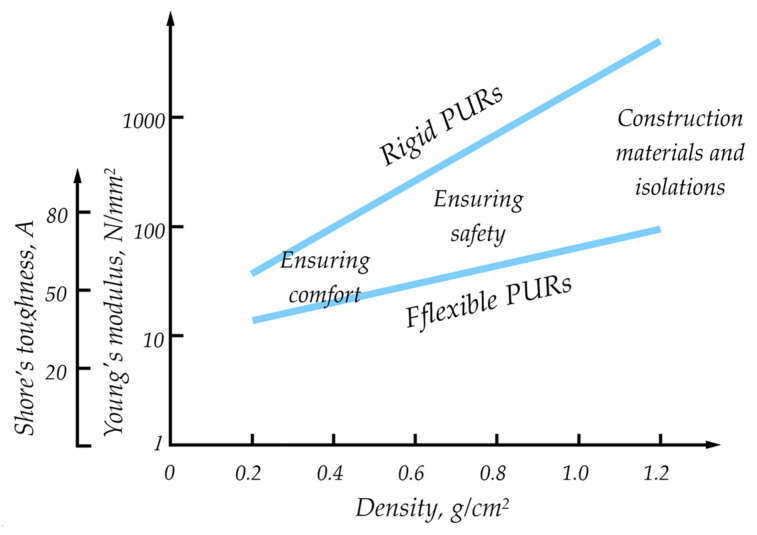
The effect of density on the physicochemical properties of polyurethanes [3].

The worldwide market for polyurethane reached a value of USD 87.10 billion in 2023 and is anticipated to increase from USD 91.49 billion in 2024 to USD 135.08 billion by the end of 2032 [6]. Due to excellent properties, there are many applications of polyurethanes, with the most popular ones including foams, coatings, adhesives, sealants, and elastomers (the last four are known as CASE), or any thermoplastic polyurethanes [7] (Figure 1). The polyurethane market exhibits a fragmented structure. Among the key participants in the market, without any specific ranking, are Huntsman International LLC (Salt Lake City, UT, USA), Wanhua (Yantai, China), Covestro AG (Leverkusen, Germany), BASF SE (Ludwigshafen, Germany), and DOW (Midland, MI, USA). In 2023, the Asia Pacific region held the largest market share, accounting for over 44.0% of the revenue. China emerged as the market leader in 2023, leading in both volume and revenue. The most popular applications are polyurethane foams (PUFs), which are divided into rigid and flexible foams. The latter dominate global foam production and are utilized in many sectors of industry [8]. When it comes to foamed polyurethane plastics, there are several divisions. The basic division is into flexible, rigid, and semi-rigid foams. They differ in cross-linking density and stiffness; these relationships are presented in Figure 2.

It is worth mentioning the wider applications of polyurethane foams, as they truly accompany humans at every step, starting from footwear soles [10]. Flexible PUF exhibits high elongation at break values and possesses desirable properties such as insulation (including acoustic and thermal properties) and good cushioning [11]. Owing to these properties, they are also present in upholstery of chairs and sofas as well as mattresses. In recent times, the popularity of so-called “memory foam”, which is foam with shape memory, and “HR foam”, high-resilience foam characterized by increased elasticity and durability, has been increasing [12]. In such forms, they are not only used in furniture-making but also in the automotive industry [13]. They constitute an ideal material for filling seats, headrests, or armrests, ensuring user comfort during rides. Additionally, they can be utilized for soundproofing engine operation due to their acoustic and insulating properties [14]. In the construction sector, their main role is building insulation implemented by rigid panels [15]. They are also used for window and door frame replacements, as well as various types of seals. Thanks to their insulating properties, they have successfully found applications in energy-saving refrigerators and freezers [16]. In addition to the previously mentioned applications, polyurethanes have successfully found their place in medicine due to their biocompatibility with the human body and non-allergenic potential [17,18]. These materials have been developed for a wide array of medical uses, serving purposes from cardiac muscle [19] to tissue engineering scaffolds [20], as well as for drug and/or gene delivery [21]. They are engineered to be either inert or biodegradable. The applications described above are just a few of the wide range of potential uses of polyurethanes. In addition to these, they can also be found in the maritime industry [22,23], decoration [24], and many others [25].

## 2. Polyurethane Foam Classification and Their Properties

The basic classification, as mentioned earlier, involves categorizing foams into flexible, rigid, and semi-rigid, but it is not the only one. Within rigid foams, it is worth mentioning PIR (polyisocyanurate) foams, which exhibit much better thermal resistance due to the presence of aromatic rings in their structure formed during the isocyanate trimerization reaction, characteristic of this type of foam [26,27]. Another classification is based on whether they are one- or two-component foams, with the difference lying in the cross-linking process. Single-component foams require moisture from the air for cross-linking (this is the main factor affecting many foam properties). On the other hand, for two-component foams, air humidity is not important, as specific catalysts are used in this case to facilitate this process. Additionally, these foams differ in their isocyanate index (NCO:OH). Within single-component foams, commonly referred to as one-part foams, several other classifications can be distinguished. Thus, depending on the type of applicator, gun foams can be indicated, which need are specialized guns for application, and straw foams, which do not require additional equipment for application, as disposable plastic is included in the set.

**Figure 3:**
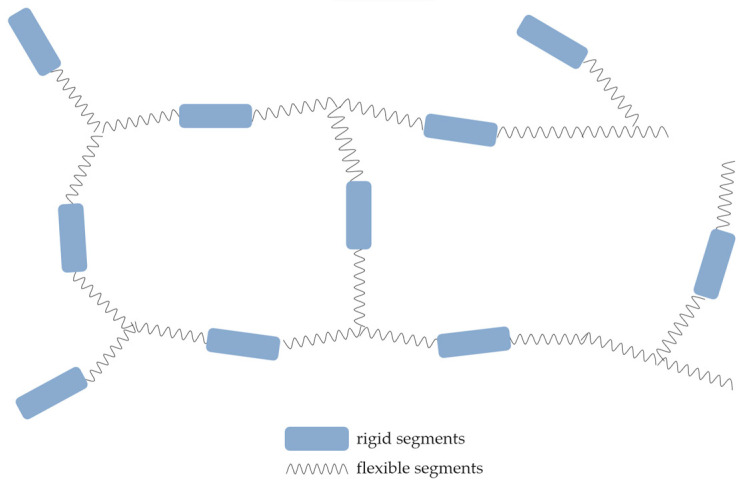
Segmental structure of polyurethanes [2].

The strength parameters are greatly influenced by the segmented structure of the polymer. As is known, the main components of such materials are polyols and isocyanates. Regarding polyols, we can distinguish two groups of raw materials with hydroxyl groups: polyethers and polyesters. The latter imparts rigidity to the polymer chain. The most commonly used isocyanates are aromatic diisocyanates terminated with NCO groups: methylene diphenyl diisocyanate (MDI) and toluene diisocyanate (TDI). The former is often used in its polymeric form, as a mixture of higher-molecular-weight MDI isomers, due to factors such as its lower cost [28]. Aromatic isocyanates contribute to the rigid segments in the polyurethane matrix. Besides the two main components mentioned earlier, a wide range of additives are also utilized in the synthesis of polyurethane foams, including catalysts, blowing agents, chain extenders, surfactants, fillers, and other auxiliary agents performing specific functions, such as reducing the flammability of the product. It is precisely the characteristics and quantity of these individual raw materials, as well as the types of bonds between them, that affect the strength properties. There is a straightforward relationship stating that the more rigid elements present, the higher the hardness, abrasion resistance, and maximum operating temperature [3]. Figure 3 illustrates the segmented structure of polyurethane foam. The rigid elements consist of isocyanate groups, urethane bonds, and urea linkages. The flexible structure is composed of oligomers, including ether and some ester bonds. The foamed plastic boasts good impermeability, hardness, and thermal conductivity coefficient (λ) [29]. It exhibits water resistance and, depending on the type, good gas diffusion, ensuring the transport of excess moisture. Additionally, it is highly facile in application and characterized by a rapid processing time [30]. 

## 3. Environmental Issues and Thermal Modernization Trends 

At the end of 2019, the European Council implemented the European Green Deal. This is a set of political initiatives covered by the European Climate Law, aimed at steering the EU towards a green transformation path. The European Union aims to reduce greenhouse gas emissions by at least 55% by 2030 and achieve climate neutrality by 2050. This includes increasing the share of renewable energy, improving energy efficiency, and implementing measures to reduce emissions from the industrial, transportation, and building sectors [31]. The EU promotes a circular economy model that focuses on minimizing waste, reusing and recycling materials, and reducing resource consumption. The construction and operation sector is one of the industries that significantly contribute to energy consumption and greenhouse gas emissions in Europe. Therefore, the European Green Deal supports all efforts aimed at decarbonizing the construction industry [32]. Additionally, many companies across various sectors, such as manufacturing, construction, energy, chemistry, and automotive, already use the Life Cycle Assessment (LCA) system to assess and improve the sustainability of their products. This method evaluates the environmental impact of a product at all stages of its life—from raw material extraction, through production and use, to disposal or recycling. It is applied based on international standards such as ISO 14040 and ISO 14044, or directives on eco-design (2009/125/EC) [33,34,35,36,37].

In recent years, there has been a noticeable increase in the number of thermal modernization projects. In Poland, financial support programs such as the Thermo-Modernization and Renovation Fund and the “Clean Air” program have significantly contributed to this growth. Further growth is expected in the near future [38]. Governmental and EU support programs will continue and even expand, encouraging more building owners to undertake modernization. Innovations in nanomaterials and smart windows may become standard in thermal modernization [39]. Additionally, increasing environmental awareness among the public and educational campaigns will drive gradual changes. Stricter regulations on building energy efficiency will accelerate the pace of these changes, forcing property owners to comply with new standards. The introduction of more stringent norms will stimulate the thermal modernization market, making it an integral part of strategies to create green cities aimed at reducing carbon footprints and improving residents’ quality of life, which are crucial in this era of technological advancement [40]. It is anticipated that all these factors will lead to increased demand for polyurethanes used in building insulation, as only good thermal insulation can prevent unnecessary heat loss and high heating costs [41]. New construction projects increasingly need to meet various environmental certifications, which confirm that a building has a minimal negative impact on the environment throughout its life cycle. The most popular of these certifications are LEED and BREEAM, with the former more commonly chosen in the American markets and the latter more popular among British and European investors. Investment funds also significantly influence the decision for certification through subsidies that require investors to have confirmations that evaluate building materials against various criteria, such as insulation efficiency, airtightness, environmental impact, durability, and their effect on reducing the overall costs of the building. Such certification lowers investment risk [42,43]. In addition to the aforementioned certificates, other known certifications include Passive House (Darmstadt, German) and DGNB (Stuttgart, German), MINERGIE^®^ (Zürich, Swiss), and HQE (Paris, French) [44]. One of the newest certifications is Cradle to Cradle^®^ (Amsterdam, The Netherlands), which focuses on products and manufacturing processes designed with sustainability and circular economy in mind. The concept involves creating products that can be fully recycled and reused at the end of their life cycle without losing material value, emphasizing the growing importance of material recycling [45,46].

So far, the polyurethane foam market has been dominated by petrochemical raw materials, but emerging environmental trends have led to the search for new solutions with reduced negative environmental impacts. Currently, natural raw materials are becoming increasingly popular; however, in the case of polyurethane foams, it is only commercially possible to use 100% bio-based raw materials in the polyol group [9,47]. At present, there is no isocyanate on the market that is entirely composed of renewable resources. However, the company Covestro (Germany) has developed an isocyanate called Desmodur eco N 7300, which contains 70% renewable carbon, primarily sourced from starch derived from non-edible plants (field corn). Unfortunately, it is dedicated to applications using hexamethylene diisocyanate (HDI) trimers, such as coatings [48]. Therefore, for PU foams, which are mainly produced from limited and non-renewable raw materials, recycling is one of the most important issues for both environmental protection and supporting sustainable development and innovation across various sectors of the economy.

## 4. Methods for the Recycling of Polyurethane Foams

**Figure 4:**
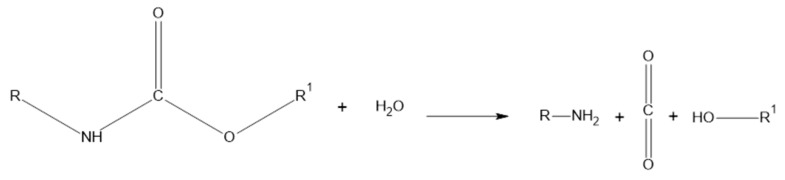
Reaction of hydrolysis.

There are many known methods for recycling polyurethane foams. The primary classification divides these methods into physical, thermal, and chemical processes [49,50,51,52] and other prospective methods, which are still under development, such as biological degradation [51,53]. Physical methods, also known as mechanical methods, involve transforming foam waste into granules, flakes, or powders that can be used as fillers, floor mats, or soundproofing mats in the automotive industry [54]. This approach is relatively inexpensive and straightforward for disposing of troublesome waste but is recommended mainly for thermoplastic polymers and not for thermoset polymers due to their behavior during mechanical processing [55]. Additionally, it can be problematic due to dust and emissions from older types of foams containing chlorinated and fluorinated blowing agents. Thermochemical and energy recovery methods focus on incineration, where the energy obtained in the process is recovered and used, for example, to generate electricity or heat [56]. Alongside physical recycling, chemical recycling is one of the most commonly used methods. It includes processes such as hydrolysis [51,57,58,59], methanolysis [60], aminolysis [61], acidolysis [62,63,64], glycolysis [65,66,67], and various hybrids of these solutions [52,68,69]. These processes are likely favored due to their technical, economic, and environmental advantages. The chemical recycling of polyurethane is feasible because its polymerization can be a reversible process under appropriate reaction conditions. During such a process, the urethane linkages break down under the influence of a suitable factor, producing monomers or basic raw materials that can be reused in manufacturing new products. Hydrolysis is one of the simplest chemical processes for recycling polyurethanes, a method implemented by Ford Motor Co. (Dearborn, MI, USA). six decades ago [70]. In this process, polyurethane foam waste is exposed to water or steam under elevated conditions within a reactor, requiring significant energy input (Figure 4). The products of hydrolysis include an amine, a polyol, and CO_2_. For example, using a CO_2_–water system as an eco-friendly reagent can break down the urethane linkages of aliphatic PU under pressure [59]. However, this method is less favored due to harsh reaction conditions, lack of markets for the recyclate, and poor economic viability. 

**Figure 5:**
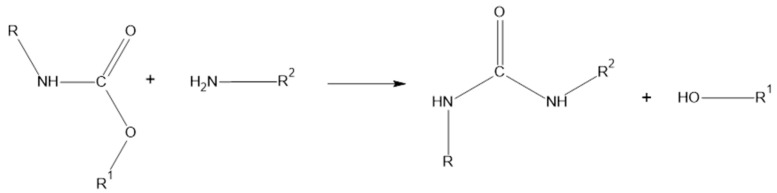
Reaction of aminolysis.

Methanolysis involves the chemical decomposition of PU wastes using methanol, similar to hydrolysis. As far as we know, Asahi et al. were the first to explore methanolysis, decomposing PUFs without a catalyst under subcritical and supercritical conditions [60]. Another method is aminolysis; appropriate to the name, it is a reaction with amines (Figure 5), the most commonly used being ethanolamine and diamines. The depolymerization process is usually carried out without an additional catalyst, because amines themselves have a catalytic effect. Unfortunately, this process has drawbacks in the form of complex side reactions, complicated extraction methods, and difficulties in separating the product [61].

**Figure 6:**
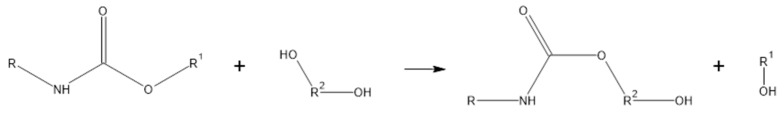
Reaction of glycolysis.

Acidolysis is another method for decomposing PU. The use of inorganic acids leads to the production of polyols, amine salts, and carbon dioxide, while organic acids, especially dicarboxylic acids, result in oligomers with urea, amide, amine, and hydroxyl groups [62]. Among the mentioned technologies, glycolysis is the most popular. In this method, the agent acting on the foam is glycol. In its presence and at elevated temperatures, with or sometimes without a catalyst, the urethane bonds break down [71]. In the glycolysis of PU foams, catalysts can be classified as homogeneous or heterogeneous, depending on whether they dissolve in the reaction medium or not. Homogeneous catalysts are preferred due to their higher activity, efficiency, better availability, and ease of use. They account for 98% of all catalysts used in this process. Examples include amines (with activity depending on their chemical structure), inorganic salts and hydroxides, inorganic acids, and phosphorus compounds. On the other hand, heterogeneous catalysts are much less common, functioning on the surface of particles, often at the phase boundary during the reaction. Examples of heterogeneous catalysts include duplex metal catalysts [72] and Zn/Sn/Al hydrotalcite [73]. Their key advantage is that they are more environmentally friendly, as they can be more easily recovered after the reaction, which suggests that they will likely be developed further in the future. The product of glycolysis is glycolysate, which can serve as a starting material for the production of new polyurethane materials [74,75,76]. The widespread use of this method is due to its low requirements and the relatively mild reaction conditions needed for its execution. It is most commonly used for recycling rigid or flexible polyurethane foams, although it has also been applied to other polyurethane materials [77]. The reaction scheme for glycolysis is shown in Figure 6; under the influence of the hydroxyl groups of the glycol, the urethane bond undergoes degradation [78].

The most commonly used glycols are short-chain glycols with low molecular weights and high polarity, such as diethylene glycol (DEG), propylene glycol (PG), ethylene glycol (EG), dipropylene glycol (DPG), glycerine, polyethylene glycol (PEG), and polypropylene glycol (PPG) [79]. When mixed in an optimal ratio glycol with foam waste and heated to a sufficiently high temperature, it results in the formation of either a single-phase or a two-phase glycolysate [80]. In the case of a two-phase glycolysate, the upper layer primarily consists of recovered polyol, while the lower layer contains unreacted glycol and undesirable by-products such as amines, aromatic carbamates, ureas, and oligomers with urethane linkages [54]. The upper phase can be directly used for the production of new polyurethane materials. However, it is sometimes further purified to concentrate the recovered polyol and reduce the amount of residual amines that affect the reactivity of the resulting polyol. The most effective method for reducing the amine content is a reaction with alkylene oxides, such as ethylene oxide (EO) and propylene oxide (PO) [81]. There are also less invasive methods, such as using epoxidized native fatty oils or adding small amounts of isocyanate, which are discussed in more detail later in this article (specifically in the patent section). The lower phase, on the other hand, constitutes process waste, rarely subjected to further treatment, but can be used for the production of rigid polyurethane foams in small quantities [82]. However, considering the economics of the glycolysis process, research is still being conducted on the valorization of aromatic compounds in the lower phase. Specifically, if the mono- and dicarbamates in the isolated lower phase can be hydrolyzed, the aromatics could be obtained exclusively as diamines, which could serve as starting materials for the production of various types of isocyanates. One method for recovering these compounds is hydrolysis with an excess of water, conducted at 200 °C for 12 h. The hydrolysis of toluene dicarbamates and aminocarbamates resulted in the formation of pentaerythritol, carbon dioxide, and toluenediamines. Vanbergen and his team performed such experiments, and the spectra obtained after the reaction were characterized mainly by signals corresponding to 2,4-toluenediamine and 2,6-toluenediamine in an 80:20 molar ratio [83]. Table 1 presents the advantages and disadvantages of the recycling methods discussed above. Over the past 30 years, the glycolysis method has been studied by numerous scientists. This article will analyze the work of some of these researchers and review the latest active patents related to recycling through glycolysis, focusing solely on polyurethane foam waste.

## 5. Glycolysis of Flexible Foam 

Flexible polyurethane foams contain certain block copolymers, whose elasticity results from phase separation between hard and soft segments. The most commonly used polyols for flexible foams are polyethers with a low hydroxyl number and a molecular weight greater than 2000 g/mol. Flexible PUFs have the widest range of applications among all polyurethanes, making their recycling an important aspect of overall plastic recycling. Table 2 presents an overview of flexible polyurethane glycolysis.

Molero et al. conducted extensive research on the glycolysis of flexible polyurethane foams. In their study, octoate salts were evaluated as novel catalysts for the transesterification of flexible polyurethane foams with diethylene glycol. The researchers found that the entire family of commercial metal octoates exhibited catalytic activity in the glycolysis process. The octoates showed different catalytic activities based on their hardness and coordination ability. Furthermore, among the octoates studied, lithium and stannous octoates demonstrated remarkable catalytic activity, resulting in the highest quality for the recovered polyol and the highest decomposition rates compared to other octoate salts. This suggests that octoate salts have the potential to outperform traditional catalysts in the transesterification of polyurethane foams, offering improved efficiency and quality in the recycling process [78]. The glycolysis of waste flexible polyurethane foam from automotive seat cushions using different industrial-grade glycols and potassium hydroxide as a catalyst was investigated by Jin et al. [84]. The choice of glycolysis agents with varying molecular weights (e.g., GPX-600, GPX-3000, GPX-6000, with molecular weights corresponding to those in their trade names) also affects the reaction conversion and viscosity of the recovered polyols. Regardless of the amount of glycolytic agents used, an increase in catalyst content results in a higher conversion rate. The optimal glycolysis condition was determined to be a catalyst concentration of 1.0 wt.%. Only with the use of low-molecular-weight glycol was a split-phase glycolysate obtained, while a single-phase recovered polyol was achieved for the other two agents. Sendijarevic from Troy Polymers Inc. (Madison Heights, MI, USA) has developed a chemical process for recovering waste polyurethane foam from shredder residue and converting it into polyols for polyurethanes. This process is ideal for recycling polyurethane foam waste from shredder residue (SR), which is a mixture of various types of PU foams. The PUFs separated from SR are contaminated with other types of non-polyurethane cellular and fluffy materials. In the first stage of this process, the PU foam undergoes glycolysis, followed by filtration of the liquid glycolysis product. In the second stage, the glycolysis products are used as initiators in the reaction with propylene oxide to produce new PU polyols. Preliminary economic analysis indicates that the commercial production of polyols from waste foam can be profitable. Aguado’s group [71] conducted research on the glycolysis process of flexible foam using diethylene glycol as the glycolysis agent in the presence of an amine catalyst, diethanolamine. The study examined three mass ratios of the catalyst to solvent (1:9, 1:6, and 1:3), three process temperatures (170 °C, 190 °C, and 210 °C), and three reaction times (30, 90, and 120 min). The research demonstrated that the optimal temperature for the process is 210 °C, as increasing the reaction temperature significantly accelerates the foam degradation. The non-catalyzed reaction results in about 50% polyol after 150 min, whereas using the catalyst achieves nearly 70% polyol in just 25 min. Using the smallest amount of DEA (DEA/DEG = 1:9) yields higher efficiency than using larger amounts, and a high concentration of the catalyst negatively affects the process by causing side reactions that contaminate the upper phase. Increasing the amount of glycol does not improve process efficiency, making the optimal mass ratio (DEA + DEG)/PU 1.25:1 [85]. Datta investigated the influence of various glycols on the chemical structure and thermal stability of glycolysates. The reaction was conducted with a mass ratio of polyurethane foam to glycols of 10:1, using a catalyst amounting to 0.5% of the polyurethane foam mass. The study reported that the type of glycol used (ranging from ethylene glycol to hexane-1,6-diol) had a negligible impact on the chemical structure of the glycolysates, as evidenced by similar absorption bands in the FTIR spectra. GPC analysis indicated that the glycolysis conditions allowed for the recovery of the original polyol, as shown by the presence of a well-defined peak at the beginning of the retention time. The study also found that the thermal stability of the glycolysates increased with the molecular weight of the glycol used. The glycolysates exhibited thermal resistance up to approximately 240–260 °C, and the temperature at which a 5% mass loss occurred was likely due to the evaporation of unreacted glycol rather than degradation [86]. Jutrzenka and her team studied the glycolysis process using refined glycerine (99.5%) and crude glycerine with purities of 84% and 62% while testing different catalysts. The best results were obtained with the dibutyltin dilaurate (DBTDL) catalyst, which ensured the highest efficiency and selectivity of the process. It was found that higher concentrations accelerate the reaction but do not significantly affect the composition of the product. Additionally, it was demonstrated that crude glycerine with 84% purity can be successfully used for the chemical recycling of flexible foam [87]. Extensive research on flexible foam was also conducted by Simón and his team. They investigated the impact of stannous octoate catalyst concentration on the reaction rate and the quality of the recovered polyol. It was found that there is an optimal catalyst concentration (1.3 wt% relative to glycol), beyond which further increases do not significantly improve the reaction rate. The researchers compared weight ratios of 0.9:1, 1.125:1, and 1.5:1, identifying an optimal ratio of 1.125:1, which provided a balance between reaction rate, polyol content in the upper phase, and the properties of the recovered polyol. It was also determined that the optimal temperature for this process is 189 °C, as it ensures a fast reaction without excessively enhancing side reactions [88]. The aforementioned author also conducted studies on the glycolysis of foams containing polymeric polyols, specifically based on polyether polyol [poly(propylene oxide-block-ethylene oxide)] with a molecular weight (Mw) of 3500. The tests revealed that the kinetics of the glycolysis reaction is slower for foams containing polymer polyols compared to foams without these polyols. However, the recovered polyol, after appropriate purification, can be reused in the synthesis of new polyurethane products [89]. In his subsequent article, the scientist explored the possibility of using crude glycerine [90], a by-product of biodiesel production, as a new transesterification agent in the glycolysis process of polyurethane foam waste. It was demonstrated that crude glycerine allowed for obtaining a glycolysis upper phase with higher purity compared to using DEG as the glycolysis agent. This was attributed to the higher dielectric constant of glycerine, which caused the by-products to be more soluble in the glycerine lower phase. Simón and his team also investigated the glycolysis of high-resilience foams (35HR) based on PIPA HR polyol [poly(propylene oxide-block-ethylene oxide)] and toluene diisocyanate. By applying previously developed process conditions, they achieved a two-phase glycolysate suitable for reuse [98]. Wu et al. tested potassium acetate as a catalyst and diethylene glycol as the glycolysis agent. After a reaction time of 90 min, the hydroxyl number, conversion of the functional –NCOO– group in PU determined by Fourier transform infrared spectroscopy (FTIR), and the average molecular weight (Mw) reached nearly constant values. The study demonstrated that glycolysis products containing polyol can be most efficiently recovered through distillation within the temperature range of 245–260 °C. The most common by-product of glycolysis was found to be methylenedianiline (MDA), which forms in the presence of water during the reaction phase [91]. Alavi Nikje and his team researched the microwave-assisted glycolysis of waste polyurethane foam. The use of microwaves significantly reduced the glycolysis time compared to conventional heating methods. For instance, at 180 °C, the reaction time decreased from 29 min to 92 s with the use of potassium hydroxide (KOH), and from 42 min to 81 s with the use of sodium hydroxide (NaOH). The NaOH catalyst proved to be a better choice than KOH. However, additional treatment did not improve the purity of the resulting glycolysates. The 1HNMR and 13CNMR spectra showed additional peaks compared to the original polyols, indicating the presence of impurities such as aromatic compounds and transesterification products [92]. Yesica Dayana Morcillo-Bolaños et al. focused on the glycolysis of PU using Zn/Sn/Al hydrotalcite as a heterogeneous catalyst. The study investigated the impact of different reaction conditions on polyol recovery and tested the recovered polyol as a partial substitute for virgin materials in the synthesis of new polyurethane foams. It was found that the addition of the Zn/Sn/Al catalyst significantly increased the amount of recovered polyol compared to the reaction without a catalyst or using NaOH as a catalyst. The resulting foams exhibited satisfactory mechanical properties even with a 10% inclusion of the recovered polyol [73]. Gabriel Kiss and his team focused on the glycolysis of flexible foams based on polyester polyols. They examined various techniques for glycolysis of polyurethane foam waste, including the atmospheric pressure method, autoclave method, and high-frequency method, evaluating the maximum possible yield of the recovered material. All three methods successfully decomposed PU foam waste, resulting in a homogeneous brown liquid with typical properties. The glycolysis using microwave equipment significantly reduced the time required to complete the decomposition, but its potential industrial application would involve increased equipment costs and high energy consumption. The autoclave glycolysis method proved to be the best among the tested techniques, allowing for increased utilization of foam waste in a single process [93]. Advanced studies were also conducted by Molero and his team. They found that diethylene glycol is the most suitable glycol for achieving high purity in the polyol phase. When using lower-molecular-weight glycols such as MEG, DEG, and MPG, a three-phase mixture was obtained. The upper phase mainly consisted of recovered polyol, the lower phase was formed from excess glycol and by-products of the reaction, and there was a small solid bottom layer originating from calcium carbonate used as a mineral filler during PU synthesis. Higher-molecular-weight glycols are likely to increase the mutual solubility of the phases or even prevent phase separation [80]. In another work, they tested the reactivity of catalysts, including two new ones (potassium octoate and calcium octoate). DEA and potassium octoate led to the fastest decomposition of the polyurethane material. Potassium octoate proved to be the most promising catalyst, as it resulted in the complete degradation of the polymer chain in a short reaction time and the recovery of polyol at a high concentration, while also demonstrating high selectivity for the glycolysis process. The amount of amines in the recovered polyols was also measured, varying depending on the catalyst used, with potassium octoate yielding the lowest primary amine content [94]. The next step was to investigate the impact of key reaction parameters on the process and the properties of the recovered polyol using diethylene glycol and potassium octoate, which were identified as the most effective variables in previous studies. Increasing the catalyst concentration shortened the degradation time of the PU foam but also reduced the purity of the recovered polyol. Additionally, it increased the amount of undesirable carboxylate salts. Raising the reaction temperature accelerated the degradation of the PU foam but also increased the degree of side reactions, thereby lowering the quality of the recovered polyol. Increasing the ratio of the glycolysis agent to PU foam improved the purity of the recovered polyol but also reduced the yield of the polyol phase. A higher amount of the glycolysis agent acted as an extracting agent for impurities, removing the side products of the reaction from the polyol phase [95]. Vanbergen et al. also conducted studies on the glycolysis process of flexible foam, focusing on identifying additives and alcoholizing agents to enhance the efficiency of the process. By testing various glycols, they found that 2-pyrrolidone positively influenced the reduction in the amount of alcoholizing agent used and increased the purity of the recovered polyol. Adding lactam to PU foam in a weight ratio of 0.1:1 to the glycolysis process significantly reduced the dissolution time of PU foam and increased the recovery yield of polyetherol. Using diglycerol as the alcoholizing agent resulted in obtaining polyol with a high purity (97%) and yield (98%) compared to other tested glycols. The polyetherol recovered using diglycerol (technical grade containing α,α-diglycerol (<90%)) and 2-pyrrolidone could be reused in the synthesis of new PU foams, replacing up to 50% of the original polyol without significantly degrading the foam quality. Additionally, studies were conducted on the lower phase of glycolysis, which typically represents an underutilized and problematic by-product. Hydrolyzing the lower phase of the alcoholysis product using pentaerythritol converted the aromatic carbamates contained in it into toluenediamines, which is a significant step towards fully utilizing all the products of PU foam glycolysis [83]. Del Amo et al. conducted analyses to determine whether the two-phase glycolysis process could be scaled up from the laboratory to a pilot scale while maintaining the properties of the recovered polyol. Additionally, the authors aimed to evaluate the economic feasibility of this process on an industrial scale. To achieve this, a pilot installation was designed (10 times larger than the lab-scale setup) using equipment with similar geometry to that used on the laboratory scale. Based on the obtained data, an industrial installation for two-phase glycolysis with a processing capacity of 270 tons of PU foam waste per year was designed. The upper phase obtained at the pilot scale consisted mainly of recovered polyol (81% by weight), with small amounts of reaction by-products and dissolved glycerol. After purification with water, a product with a purity of >96% by weight of recovered polyol was obtained. It was demonstrated that this process is technically feasible on a larger scale. The calculated net present value and internal rate of return confirmed that this process is profitable and can yield significant profits. The estimated payback period for the investment was between 4 and 5 years [96]. Pang and his team focused on foam derived from upholstered furniture. In their research, they used propylene glycol (PPG) as a solvent and sodium hydroxide (NaOH) as a catalyst. The hydroxyl number and amine number increased as the reaction progressed, while the viscosity decreased. The recovered polyol obtained at 200 °C and a PPG/PU weight ratio of 3:1 exhibited similar reaction time and physical properties to the polyol obtained at 180 °C and a PPG/PU weight ratio of 4:1. At the PPG/PU weight ratio of 4:1, phase separation of the glycolysate was observed, suggesting that excess PPG may lead to a biphasic product. FT-IR analysis confirmed the breakdown of urethane bonds in the PU foams during the reaction, as evidenced by the disappearance of the peak characteristic of the urethane carbonyl group. The studies discussed above clearly indicate that the glycolysis of flexible polyurethane foam most often leads to a split-phase glycolysate, where its lower layer constitutes a process waste rarely subjected to further treatment [82]. Peng and his group focused on developing an eco-friendly degradation process for waste polyurethane foam that would allow full utilization of the decomposition products without advanced purification and separation methods. The triblock polyether (Triblock Polyoxypropylene Polyoxyethylene Copolymer, PEG-PPG-PEG) L31 (Mn = 1100 g/mol; functionality: 2) and polyether polyols (based on PPG) HSH-210 (Mn = 1000 g/mol; functionality: 2) were used as glycolysis agents. It was found that reaction time, the weight ratio of L31 to PU foam, and the type of glycolysis agent used had a significant impact on the efficiency of PU foam degradation. L31 showed significantly higher reactivity compared to HSH-210, which allowed for the obtainment of single-phase products at a lower molar ratio of glycolysis agent to PU foam. This product was used to synthesize new PU foam, which exhibited good elasticity, porosity, and an open-cell structure, making it suitable for oil absorption applications. As seen, it is possible to obtain both single-phase and two-phase products when flexible polyurethane foams undergo glycolysis [97].

## 6. Glycolysis of Rigid Foam

Rigid PUFs arehighly cross-linked polymers and, unlike flexible PUFs, -are produced from polyols with a low molecular weight below 1000 g/mol. The polyol base consists of both polyethers and polyesters. The nature of the polyol and the category of hydroxyl groups in the polyol directly influence the physical and mechanical properties of the produced PU. Due to their low thermal conductivity and good mechanical properties, they are used as thermal insulation materials in various applications as well as sound-absorbing materials. Their wide range of applications generates large amounts of waste, which is a concerning issue; therefore, their management has become an important matter [99]. The following Table 3 presents an overview of rigid polyurethane glycolysis.

Research on the degradation rate of rigid polyurethane foam under different reaction conditions was conducted by Michio Murai and his team. They investigated various polypropylene glycols, polyethylene glycols, diols with different chain lengths, and high-functionality polyols. The influence of different catalysts and temperatures on the decomposition rate of selected foams was also examined. Additionally, the dissolution time of PUF blocks of different sizes was compared. The tests showed that dipropylene glycol and tetraethylene glycol dissolved PUF in the shortest time among the tested polypropylene glycols and polyethylene glycols. High-functionality polyols, typically used for producing rigid polyurethane foams, resulted in longer dissolution times compared to those with lower functionality and simpler chains. The dissolution time of PUF decreased by half with every 10 °C increase in temperature within the range of 170–200 °C. Alkali metal hydroxides, especially KOH, proved to be the most effective catalysts for PUF glycolysis. Increasing the weight ratio of glycol to PUF from 1.3:1 to 2:1 enhanced the degree of PUF glycolysis [100]. Zhu et al. conducted an in-depth study on rigid foam derived from refrigerators. The results of this investigation showed that ethylene glycol proved to be a more effective glycolysis reagent than diethylene glycol due to its smaller steric hindrance. NaOH demonstrated the highest catalytic efficiency compared to triethanolamine and sodium acetate, enabling complete degradation of the rigid polyurethane foam and forming a homogeneous glycolysis product. However, an excess of NaOH negatively impacted the process. Another type of rigid foam is PIR foam, which contains isocyanurate rings in its structure, making it more thermally stable [101]. Michele Modesti and his team worked on its chemical recycling process, aiming to determine the optimal conditions for such a process. They demonstrated that despite its high thermal stability and cross-linked structure, it is possible to recover polyols through glycolysis. They chose dipropylene glycol as the glycolysis agent due to its high reactivity, as well as its lipophilic nature and suitability for forming a monophasic product because of its secondary hydroxyl end-groups. Potassium acetate proved to be a more suitable catalyst for the glycolysis of PIR foams compared to titanium(IV)-n-butoxide (TNBT). Using TNBT required higher temperatures and concentrations, making the process less efficient. The optimal PU/glycol ratio was 1:1.5. Increasing the reaction temperature accelerated glycolysis but also increased the degree of side reactions, such as pyrolysis and hydrolysis. Glycolysis products obtained under optimal conditions, when combined with virgin polyol, allowed for the production of PUR and PIR foams with mechanical properties and cellular structure comparable to, or even better than, foams produced exclusively from virgin polyol [102]. Gu et al. investigated the impact of different catalysts on the glycolysis process of rigid foam waste from refrigerators. In their experiments, they used a mixture of two glycols in an EG:DEG ratio of 6:4. Titanium glycolate proved to be a more effective catalyst than KOH under most conditions studied. The obtained glycolysate was used in the synthesis of new rigid foams. SEM analysis showed that the foam produced using titanium glycolate had a more uniform cellular structure compared to PUF obtained using KOH, and it also exhibited better mechanical properties [103]. In different work, they investigated the impact of an innovative self-made core–shell nanoscale titanium catalyst compared to a traditional alkaline catalyst (KOH). The titanium catalyst demonstrated generally higher catalytic efficiency compared to KOH, as evidenced by the lower viscosity of the recovered polyether polyol at lower catalyst concentrations. Using glycolysate with the titanium catalyst resulted in PU foams with higher compressive strength compared to PU foams produced using KOH. However, for both catalysts, the values of water absorption and thermal conductivity were comparable [104]. In another study, Gu et al. researched the conditions of different catalytic degradation systems. They catalyzed the reactions using duplex metal catalysts (DMCs) and alkali metal catalysts, and also explored the synergy between the two. The DMC was a compound primarily composed of Zn and Co ions, with the high catalytic activity mainly attributed to the Co ions. It was found that the synergistic catalytic system of NaOH and DMCs was the most effective for the degradation process of waste PU foam. The optimal conditions for the degradation process were determined to be 0.25% NaOH, 0.04% DMC, a reaction temperature of 160 °C, and a reaction time of 2.5 h. The most effective glycolysis agent mixture was found to be an EG:MPG ratio of 60:40. The PU foam produced using the recovered polyol exhibited high compressive strength, good thermal stability, and a uniform cellular structure [72]. Another work by Gu examined the optimal conditions for the glycolysis process using a mixture of glycerol and butanediol as the alcoholysis agent while testing the effectiveness of cesium hydroxide (CsOH) at concentrations of 0.04%, 0.06%, 0.08%, 0.10%, and 0.12%, and KOH at a concentration of 0.5%. The optimal parameters were found to be a GLY:BDO ratio of 3:2, a PU to glycol mixture ratio of 1:1.2, 0.08% CsOH based on the total amount, and a reaction conducted at 170°C for 2.5 h. Using CsOH as the catalyst resulted in significantly better degradation outcomes compared to the traditionally used KOH. The recovered polyol obtained with CsOH exhibited lower viscosity, a hydroxyl number closer to that of commercial base polyether polyol, lower molecular weight, and a narrower molecular weight distribution. This recovered polyol was used to create new thermosetting polyurethane rigid foam, which had a density of 34.1 kg/m^3^ and a compressive strength of 0.301 MPa, yielding satisfactory results [105]. Riccardo Donaldini’s research team explored the potential of using microwaves to enhance the speed and efficiency of glycolysis of rigid PU foam compared to traditional conventional heating. Additionally, they assessed the impact of three selected catalysts: potassium acetate, tin(II) 2-ethylhexanoate or stannous octoate, and monoethanolamine. The use of microwaves reduced the reaction time by 94% compared to conventional heating while also reducing energy consumption by 45%. Both KAc and Sn(Oct)2 yielded glycolysates with low viscosity, indicating a high degree of PU foam depolymerization. However, KAc promoted the formation of more by-products, such as methylenedianiline. The glycolysate obtained with KAc at a concentration of 30 mmol/100 g PU exhibited the best properties for producing new PU foam. The PU foam produced with up to 30% glycolysate showed better mechanical properties (compressive strength) and comparable thermal properties (thermal conductivity) compared to foam produced using only virgin polyol [106]. Rafael Miguel-Fernández and his team investigated the depolymerization of two types of rigid foam (based on aliphatic and aromatic isocyanates, respectively) through glycolysis. Aromatic isocyanate-based PU underwent glycolysis with a high conversion rate (>90%) within 2 h, even without a catalyst. In contrast, aliphatic isocyanate-based PU required the use of catalysts. NaOH and sodium acetate effectively catalyzed the glycolysis reaction, with NaOH providing a more stable reaction course. However, DEA proved ineffective, reducing the conversion percentage. Among the two tested solvents, EG offered better properties; the resulting products had lower viscosity and were easier to clean (filter and centrifuge). During the creation of new foams, the recovered polyol performed better in formulations with aliphatic isocyanates due to the lower overall reactivity of the system, as the recycled polyols exhibited very high reactivity. Consequently, it was proposed to use the recycled polyol as a substitute for the commercially used catalyst DBTL in the production of rigid polyurethane foam [107]. Izotz Amundarain and colleagues focused on foams with recycled polyol derived from the glycolysis of rigid foams used in housings and brackets. The recovered polyols had higher hydroxyl numbers, acid numbers, average molecular weights, and viscosities compared to commercial polyols due to the presence of glycolysis by-products (carbamates, amines) and residual EG used in the process. FTIR analysis revealed the presence of C=O (1737 cm^−1^) and N-H (1514 and 1614 cm^−1^) bonds of urethane and urea groups in the recovered polyols, indicating limitations in the PU glycolysis reaction. Adding the recovered polyols to the formulation of new RPUF foams accelerated the foaming reaction, as evidenced by shorter characteristic times and more exothermic temperature profiles. Moreover, the tensile properties of the resulting foams significantly improved, likely due to the greater cross-linking in samples containing the recycled polyol [108]. Maioli et al. investigated the degree of polyurethane conversion during the glycolysis of rigid foam. They tested the influence of two glycolysis agents (glycerin and diethylene glycol) and various catalysts, such as NaOH, ionic liquids (LIMn, LIZn), and other previously unused catalysts. The conversion rate of PU to the product was calculated based on 1H NMR spectra by analyzing aromatic proton signals. Using NaOH as a catalyst in GCL allowed for 100% PU conversion to the product in less than 30 min. Ionic liquids LIMn and LIZn also showed good catalytic activity in GCL, achieving conversions of approximately 80% within 1 h and 70% within 1.5 h, respectively. The reaction without a catalyst using glycerin also showed significant PU degradation, likely due to the high reactivity of GLY resulting from the presence of three hydroxyl groups, whereas the use of DEG negatively impacted the depolymerization efficiency. DOSY-NMR analysis proved helpful, revealing a complex mixture of by-products in the depolymerization reaction, contrasting with 1H NMR data that suggested the formation of a simple diamine-terminated by-product [109]. Donaldini et al. also assessed the performance of three deaminating agents: an epoxy (2-(2-ethylhexoxymethyl)oxirane also known as 2-ethylhexyl glycidyl ether (2-EHGE)), an anhydride (acetic anhydride (Ac2O)), and 1,3-dioxolan-2-one also known as ethylene carbonate (EC) to decrease the MDA content in recycled polyol via glycolysis. The amount of resulting MDA was influenced by the concentration of the catalyst and the reaction time; higher concentrations of KAc and longer process times led to higher MDA concentrations. The catalyst potassium acetate in the amount of 30 mmol/100 g of polyurethane proved to be the best solution, ensuring a high conversion rate and low levels of the undesired component. Among the tested deaminating agents, 2-EHGE was the most effective, providing almost complete conversion of MDA in less than 15 min, regardless of the reaction temperature. Additionally, tests of the glycolysis product in PU foam showed a positive result in the form of improved compressive strength [81].

In summary, the glycolysis of rigid polyurethane foams most frequently involved foam from refrigerators. The most commonly used glycolysis agent was diethylene glycol, while potassium acetate was the preferred catalyst. The recycled polyol was predominantly monophasic and successfully utilized in the synthesis of new rigid foams, albeit only on a laboratory scale. An increase in the mechanical strength of materials containing glycolysate was generally observed. Currently, the available literature does not contain information on the recycling of semi-rigid polyurethane foams through glycolysis. It is worth noting that these foams also pose a problem and are widely used in construction and other industries.

## 7. Patented Technologies for the Glycolysis Process

In the earlier part of this article, an exhaustive review of the literature was presented concerning the glycolysis process of polyurethane foams. However, in most cases, the studies and obtained results pertained exclusively to the laboratory scale. Techniques for chemical recovery are challenging to implement on an industrial scale. Currently, the only company globally that has achieved industrialization of degradation methods is H&S Anlagentechnik GmbH from Germany [110]. While they have developed equipment and a process involving a single reaction tank, the equipment and process still struggle with effectively addressing mass transfer and heat transfer issues during foam degradation, and they require an exceptionally long reaction time. The topic of recycling has been known for a long time, resulting in the publication of many patents in this field. Patents provide an invaluable knowledge base that is worth examining.

The first patents related to the glycolysis of polyurethane foams emerged in the 1970s. At that time, patent US3738946A was published by UPJOHN US, which proposed the thermal treatment of polyurethane waste at a temperature range of 175–250 °C in the presence of a dihydroxy compound consisting of 90–95% by weight of an aliphatic diol containing two to six carbon atoms (with a boiling point above approximately 180 °C) and 5% to 10% of a dialkanolamine by weight [111]. Another similar patent, describing the basic glycolysis process, is numbered US3983087A and belongs to The Dow Chemical Co [112]. In 1987, McDonnell Douglas Corp. published a patent addressing the high reactivity of recycled polyol, attributing it to the presence of amine groups. The patent proposes a reaction with alkylene oxide, after the addition of a solvent (with a boiling point above 120 °C) to reduce the viscosity of the PU degradation product mixture. The selected solvents are amines containing at least one primary or secondary amine group, which can be either aliphatic or aromatic amines [113]. In the 1990s, there was an increase in patents related to the glycolysis process. In 1994, Air Products & Chemicals, Inc. published the patent US5300530A, which focused on the modification of the obtained glycolysate. The process involves converting polyurethane foam into a reusable polyol composition by glycolyzing to produce a glycolysis polyol containing amines. This product is then reacted with an alkylene oxide in a ratio of less than one mole of alkylene oxide per mole of active amine hydrogen atoms present in the glycolysis polyol. This reaction between primary amines and alkylene oxide lowers the concentration of primary aromatic diamines to below 0.1% and decreases the reactivity of the glycolysis polyol product. However, the remaining secondary amines enable the polyol to still function effectively as a cross-linker in polyurethane formulations, enhancing the compressive strength of the final polyurethane product [114]. In the same year, Basf Schwarzheide GmbH also published its patent for the production of recycled polyols with a low amine content. Their idea involved reacting polyurethane waste with short-chain compounds containing at least two OH groups in the presence of catalysts, which includes dosing mono- and/or difunctional glycidyl ethers throughout the reaction process. The glycidyl ethers are added in an amount ranging from 5 to 20% by weight, based on the total reaction mixture. The preferred reaction temperature is 200–235 °C, and the process should be conducted for 3 to 5 h [115]. Another patent from the same company, this time issued for the Korean market as KR1019960022720A, describes a process where the recovered polyol reacts with at least one epoxidized natural fatty oil at a temperature of 130–180 °C. The epoxidized natural fatty oil is added in an amount of 2–10% by weight, based on the total weight of the polyurethane and the glycol used [116]. The equivalent of this patent for the United States was issued two months later [117]. In 1997, HUNTSMAN ICI CHEM LLC (The Woodlands, TX, USA) published its concept for recycling flexible foam using low-molecular-weight polyols, followed by allowing the mixture to separate into two phases, and then using extraction. The glycolysis process was carried out between 170 and 240 °C for 2 to 8 h, using ethylene glycol or diethylene glycol. This process results in two phases, with the upper phase, rich in polyol, undergoing extraction. In this case, the extracting agent is a polyol or a polyol mixture with an average molecular weight of at most 500, and being immiscible with the polyol. Importantly, the extraction process is conducted 2–10 times, either batchwise or continuously [118]. Even Samsung Electronics Co., Ltd. (Suwon, Republic of Korea) filed a patent application in the late 1990s, describing a glycolysis process. In this process, a mixture of 100 parts PU scrap, 15–100 parts glycol, and 0.01–10 parts catalyst (sodium hydroxide) is placed into a reaction and allowed to react under a nitrogen atmosphere at a temperature of 120–300 °C for 0.5–15 h, resulting in recycled polyol. Importantly, the polyurethane foam scrap had an average diameter of about 80 μm or less [119]. Another patent issued by BASF Corp. concerns the production of glycolysate with a low amine content. This time, to reduce the amount of primary aromatic amines, the addition of a cyclic carbonate was proposed (added in an amount of about 5–30% by weight of the polyurethane component) [120]. In 2000, Basf Schwarzheide GmbH focused on modifying the glycolysis agent. Alongside the usual low-molecular-weight glycol, they also used a polyether polyol. Depending on the type of foam, a different polyether was selected: for flexible or semi-rigid polyurethane foams, they chose one with a hydroxyl number of 20 to 120 mg of KOH/g. For rigid foam, they recommended using a polyol with a hydroxyl number of 200 to 800 mg of KOH/g [121]. In contrast, a patent issued in 2003 by ТУРЧЕНКО ДМИТРО КУЗЬМИЧ, valid in Ukraine, describes a glycolysis process that uses wastes from polyethylene terephthalate, polyurethane foams, and vegetable oils in a single process [122]. Troy Polymers, Inc. developed a method that utilizes chemolysis products as initiators in reactions with propylene oxide and/or ethylene oxide to produce novel polyols with varying equivalent weights for different applications, including flexible foams, semi-rigid and rigid foams, elastomers, coatings, adhesives, and sealants. These polyols are unique due to the distinctive composition of the light automotive shredder residue (ASR), containing various aromatic groups, ether, and ester linkages. The catalysts used in glycolysis can also catalyze the propoxylation/ethoxylation process, eliminating the need for additional catalysts in the synthesis of these innovative polyols. The polyols obtained in this way can be the sole raw material forming the polyol base in the production of new polyurethane materials [123]. A patent application filed by Repsol Química SA described a process for recovering high-quality polyols from flexible polyurethane foam waste through a two-phase glycolysis process using a novel catalyst based on naphthenic salts or a mixture of their components (selected from the group of alkali and alkaline earth salts). By using a large excess of the glycolysis agent (PU/glycols = 1:1.5), the reaction product separates into two phases: the upper layer consists mainly of the recovered polyol from PU, while the lower layer contains the excess glycol. The upper phase can be purified through liquid–liquid extraction using an aqueous phase, and the excess glycol from the lower phase can be recycled back into the process via vacuum distillation. The residual phase left after distillation can be combined with propylene oxide to generate a polyol [124]. The patent application from LAMBDA ONE ISOLIERTECHN involved combining glycolysis with aminolysis. Specifically, the process for producing polyol from waste rigid polyurethane foam entails reacting the foam with a mixture of one or more glycols and one or more aliphatic amines, along with a mixture of tall oil fatty acids (10 to 40% by weight, including linoleic acid, conjugated C18 fatty acids, oleic acid, octadeca-5,9,12-trienoic acid, and saturated fatty acids) or a rapeseed oil fatty acid mixture (comprising palmitic acid, stearic acid, oleic acid, linoleic acid, linolenic acid) [125]. Polymer Recycling Technologies Ltd. has worked on enhancing the efficiency of polyurethane foam recycling. They focused on the grinding process of waste foam. By employing a wet grinding technique, their invention minimizes foam loss during grinding and increases the efficiency of chemical recycling. Additionally, this method improves the yield of the chemical recycling process by approximately 10 to 15% compared to conventional methods, leading to better overall efficiency and reduced safety concerns. The process uses recycled polyol as a wetting agent, which is introduced into the mill in a countercurrent flow, creating a mist that prevents the escape of fine polyurethane dust [126]. Among the most recent patent applications that are not yet in effect are three patents with the following numbers: WO2024081090A1, US20240239985A1, and WO2024165638A1. The first one, owned by Stepan Co., describes a process for the chemical recycling of rigid polyurethane and polyisocyanurate foams. As a chemolysis reagent, they propose a difunctional aliphatic acid (10–50 wt%) combined with a low-molecular-weight glycol (50–90 wt%), creating a mixture with a hydroxyl value of 400 to 900 mg KOH/g. Hydroxides or acetates were used as catalysts in the process. When reacting with rigid polyurethane or polyisocyanurate (PIR) foam, the result is a first polyol containing up to 55 wt% recycled foam, with a hydroxyl value of less than 550 mg KOH/g and a free glycol level of less than 35 wt%. This polyol can be used to produce new PIR foam [127]. The next patent belongs to CHEN YU TING. They focused on improving the glycolysis process by enhancing energy efficiency and addressing challenges in recycling. The proposed solution involves several steps. The first step is to mix polyurethane waste (and also polyethylene terephthalate waste) with a preheated glycolysis agent at 60–80 °C to form a premix. This premix is then heated to 180–240 °C, resulting in a liquefied mixture, which is subjected to degradation. Afterward, the polyol mixture is combined with a second glycolysis agent and undergoes a heat transfer process to produce a cooled polyol mixture, along with a second preheated glycolysis agent [128]. The most recent patent, revealed on 15 August 2024, belongs to PolyTech AS and involves the recovery of high-quality polyols from polyurethane wastes using a three-phase glycolysis process. The glycolysis agent is polyethylene glycol with a molecular weight between 380 and 420 g/mol. The catalyst was selected from metal acetate salts (e.g., zinc acetate) and used in an amount of 2 to 4 wt.% relative to the polyurethane used. The ratio of PU to glycol was set at 1:1 or 1:2, and the process temperature ranges between 180 °C and 220 °C. After the decomposition process, which can last up to 240 min, the mixture is separated into at least three immiscible phases to obtain a recovered polyol phase, a glycolyzing compound phase, and a waste phase [129].

The next patents that were thoroughly analyzed are those related to the glycolysis of PU foams and are currently active. This section provides an overview of these patents, with Table 4 summarizing the findings.

The invention described in ES2277554B1 pertains to a method for creating polyols from the recycling and/or reuse of rigid polyurethane and/or polyisocyanurate foam materials via glycolysis. Subsequently, the glycolysis products are treated with a blend of organic acids to minimize the presence of unwanted primary aromatic amines in the final product. The resulting polyols can be utilized to manufacture new rigid polyurethane, polyurea/polyurethane, or polyisocyanurate foams. Short-chain polyols typically contain two to three hydroxyl groups, such as DEG or PG. The process of the present invention can utilize known and conventional catalysts, including alkali metal salts of short-chain fatty acids, potassium hydroxide, sodium hydroxide, and organic titanium salts. Suitable organic acids for this invention include carboxylic and dicarboxylic acids. Examples of carboxylic acids are acetic, propionic, butanoic, and pentanoic acids, and their higher homologues, as well as mixtures of these. Particularly preferred are organic acids with a boiling point above 150 °C, such as hexanoic acid, octanoic acid, and their homologues, as well as mixtures of these. Dicarboxylic acids include oxalic, malonic, succinic, glutaric, adipic, maleic, and fumaric acids, and anhydride-type diacid precursors like maleic anhydride or phthalic anhydride, along with their mixtures. In this invention, the organic acid is generally added to the reaction mixture in an amount ranging from 5% to 80%, preferably between 10% and 20% by weight relative to the polyurethane residue in the reaction mixture. The resulting regenerative polyols were characterized by a low MDA content but a high hydroxyl number, above 560 mg KOH/g [130]. In patent KR101061839B1, the novelty lies in the use of pentaerythritol or sorbitol along with a low-molecular-weight diol. This approach allows the glycolysis of foam to proceed with adequate efficiency while also producing a higher functionality recycled polyol, which benefits subsequent applications. Often, foams created using recycled polyols exhibit shrinkage. The use of a higher-functionality glycolysis agent increases the overall functionality of the recovered polyol, resulting in foam that does not shrink and has enhanced compressive strength [131]. In patent EP2183311B1, microwave heating and a low-molecular-weight polyhydric alcohol were utilized. This process allows for faster decomposition of the foam and reduces energy consumption. The reaction mixture is heated using electromagnetic radiation with frequencies ranging from 1 MHz to 10 GHz, reaching temperatures between 50 °C and 300 °C. Waste polyurethane foams are recycled into a mixture of polyols through glycolysis, which is catalyzed by adding monoethanolamine, diethanolamine, triethanolamine, sodium or potassium hydroxide, sodium or potassium ethanolate, titanium butoxide, isopropoxide, propoxide, or their mixtures in any ratio, with a catalyst to polyurethane foam weight ratio of 1:500 to 1:10. This microwave-based glycolysis technology is energy-efficient, reducing electricity consumption by up to 70% compared to traditional glycolysis methods [132]. Another patent US8609740B2 states that to make the recovered polyol processable, a viscosity reducer, such as a dibasic ester at a level of 1–10 parts by weight, can be added. The dibasic ester(s) can be purified dimethyl esters of adipic, glutaric, and succinic acids. Thanks to this addition to the recycled polyol, the foam waste became a 100% usable raw material in the production of new PU foams, enabling the implementation of a circular economy [133]. Another patented solution involves the addition of an alcoholysis aid during the glycolysis reaction stage. The alcoholysis aid is chosen from two groups: the first group consists of N-methylmorpholine, phosphite, and carbodiimide, while the second group consists of butylated diphenylamine, octylated diphenylamine, and carbodiimide. The process should be conducted at 150–200 °C for 5–10 h. The main advantage of this solution is that it allows the processing of various types of polyurethane materials, not just foams [134]. H&S Anlagentechnik GmbH has patented a method for creating isocyanate-reactive polyols from polyurethane waste, which can be utilized in the production of rigid polyurethane foams. The polyurethane waste is first reacted with a reaction mixture containing at least one dicarboxylic acid or dicarboxylic acid derivative and at least one polyether polyol with an average molar mass of 400 to 6000 g/mol and a hydroxyl functionality of 2 to 4, and preferably at least one free-radical former suitable for initiating free-radical polymerization (such as an inorganic peroxide, added to initiate or accelerate the reaction with carboxylic acids). The next step involves reacting the resulting mixture again with at least one short-chain diol or triol with two to eight carbon atoms at temperatures from 180 °C to 230 °C, preferably from 195 °C to 220 °C, to produce an isocyanate-reactive polyol dispersion. Another aspect of the invention is the use of the resulting polyol dispersion to produce rigid polyurethane foam materials, where the recycled polyol is best used in combination with a base polyol (synthetic, non-recycled) in a ratio of 10:90 to 40:60 [135]. Patent CN113429540B suggests employing a two-component alcoholysis agent along with an alcoholysis assistant to degrade waste polyurethane foam, resulting in a polyol degradation product. The reaction temperature of the degradation reaction is 130–220 °C, and the reaction time takes 1–5 h. The recycled polyol is then combined with isocyanate to produce a polyurethane thermal insulation material. The alcoholysis assistant is added to aid in the formation of a cross-linked structure in the polyurethane. The polyol products are cost-effective, high-performing, and environmentally friendly. Additionally, the resulting rigid polyurethane foam exhibits superior thermal insulation properties, with a closed cell rate of approximately 90%. Its thermal conductivity, compressive strength, apparent density, and water absorption all exceed national standards [136]. The other patent from Korea claims a method for adjusting the reactivity of the recovered polyol. The method of the invention includes two steps: a first step of introducing 50 to 150 parts by weight of glycol, based on 100 parts by weight of rigid polyurethane foam scrap, to initiate the glycolysis reaction, thereby obtaining a primary liquefied depolymerized product; and a second step of introducing 3 to 200 parts by weight of an aldehyde compound, based on 100 parts by weight of the primary liquefied depolymerized product obtained in the first step. This process allows a relatively highly reactive compound to react with the aldehyde compound to gradually decrease the reactivity of the highly reactive compound, thereby obtaining a new recycled polyol with adjusted reactivity. As is known, amine compounds have higher reactivity than others, making it difficult to control the reaction of urethane or urea formation. The application of a two-step recycling process leads to the formation of an imine or enamine, which is relatively unreactive with isocyanates. This results in a product with reduced reactivity, which is desirable during the formation of new polyurethane material [137]. Another patent, No. KR1020230041371A, from the same company also addresses reducing the reactivity of the recovered polyol. However, this time, the polyol from glycolysis, realized by adding 50 to 150 parts by weight of glycol to 100 parts by weight of rigid PU scraps, is mixed with isocyanate (50 to 150 parts TDI, MDI, or polymeric MDI to 100 parts recycled polyol). The last step involves dispersing the mixture in a new polyol to obtain a recycled polymer polyol (25 to 100 parts by weight of the glycolysis product obtained in the previous steps to 100 parts by weight of new polyol used to produce rigid foams). This procedure allows for the production of recycled polymer polyol, from which high-quality new rigid polyurethane foams can be stably and efficiently manufactured [138]. Chinese patent CN113354863B proposes a method in which waste polyurethane foam is degraded into liquid polyol by three different alcoholysis agents and an alcoholysis aid. The first alcoholysis agent is from the group: glycols, polyether polyols, and polyester polyols. The second agent is a cyclic alcohol, and the third is one from the group of glycerine, trimethylolpropane, pentaerythritol, xylitol, sorbitol, and sucrose. The pro-alcoholysis agent is one or more alcohol amine compounds, amine compounds, inorganic strong bases, and organic titanium compounds. Preferably, the temperature of the degradation is 130–220 °C, and the reaction time is 1–5 h. The invention provides a thermal insulation material consisting of recycled polyol and isocyanate. This material has excellent thermal insulation performance with a closed-cell porosity of greater than 90% [139]. The last of the analyzed patents, published in February 2024, contains a method and device for degrading and recycling waste polyurethane foam. The process flow involves the following steps: mixing polyol, degradation agent I, and catalyst for preheating; adding the mixed degradation liquid and crushed foam into preliminary reaction tank I for primary degradation to obtain the primary degradation liquid; and adding the primary degradation liquid, degradation agent II, and catalyst II to secondary reaction tank II for secondary degradation, and obtaining the final product after 1 to 3 h of reaction. The recycled polyols can be directly used for foaming. The optimized degradation process flow and equipment solve the issues of mass and heat transfer during foam degradation and adjust the efficient depolymerization of key chemical bonds in polyurethane foam. The reaction conditions are mild, and the reaction time is short, which reduces energy loss. The mass ratio of polyol, degradation agent I, and catalyst I in the activation reaction tank is 100:10:1 to 100:30:5. The polyol in the activation tank is a commercial polyether polyol, with a trade name CARADOL SC56-23. Degradation agent I in the activation tank can be one of triphenylphosphine, triethylamine, 2,2′-dihydroxydiethylamine, sodium hydroxide, sodium carbonate, citrate, acetate, phosphate, or a mixture of two or more. Catalyst I in the activation tank is selected from a group including nano-copper, nano-zinc oxide, nano-cesium oxide, nano-zinc, sodium ethoxide, sodium methoxide, zinc acetate, aluminum acetate, and arginine. The degradation agent II in the secondary reaction tank II is one or two of the following: acetic acid, citric acid, malic acid, tartaric acid, oxalic acid, benzoic acid, salicylic acid, oxalic acid, glutaric acid, adipic acid, and hydrochloric acid. Catalyst II is compounded with copper as a matrix and two metal oxides. To summarize, this invention combines glycolysis with acidolysis using a dedicated device for conducting the recycling operation. The resulting product is a 100% recyclable material, and the production technology used does not cause environmental pollution [140].

To summarize the analyzed active patents, a noticeable trend emerges where inventors focus not solely on the glycolysis process itself, the catalysis of the reaction, or enhancing the process efficiency, but rather on minimizing undesirable properties of the glycolysate, such as high amine content and high acid number. The high amine content affects the reactivity of the resulting polyol, significantly increasing it, which creates issues in later stages when using the polyol to produce new polyurethane materials. On the other hand, a high acid number impacts both the lifespan of the polyol and, later on, the preparation of component A, which includes not only the recycled polyol but also catalysts, silicones, blowing agents, and other additives, as well as the lifespan of the final polyurethane product.

## 8. Summary and Future Trends

Polyurethanes (PUs) are polymers gaining significant attention in both industry and research due to their exceptional properties. However, their widespread use has led to substantial waste, contributing to environmental pollution. Consequently, recycling PUs has become a pressing task to produce high-quality glycolysates and reduce post-consumer PU waste. Glycolysis is regarded as the most critical chemical method for recycling PUs to obtain products with good properties and high purity. As a result, it has been determined that a detailed analysis of the literature on this topic is highly important and could significantly contribute to the advancement of recycling these materials. In this context, numerous research groups have dedicated efforts to developing various reactions involving flexible and rigid polyurethane foam (PUF) and alcoholysis agents, which were discussed here. What distinguishes this article from others is its review of the latest literature and analysis of active patents, which together provide a comprehensive overview of the current state of knowledge in this field, as well as the patented solutions that are not feasible for implementation. Many variables influence the foam degradation process via glycolysis, including reaction time, catalyst, type of glycol, temperature, and PU mass ratio, and all of them have been thoroughly analyzed.

The catalyst plays a crucial role in the glycolysis process, as it significantly affects the rate of the depolymerization reaction and, consequently, the formation rate of undesirable by-products. The most commonly used catalysts in the degradation of PU foam can be divided into four groups: hydroxides, acetates, organometals, and amines. If we look at flexible foams, the most commonly used solutions were hydroxides (KOH and NaOH), KAc, and Sn(Oct)2, and in the amine group, diethanolamine. Interestingly, the optimal ratio of catalyst, which was commonly repeated in studies, was 1% catalyst to PU. No significant benefits were observed with higher amounts. When it comes to the PU ratio, the most effective combinations are 1:1, 1:1.5, and 1.5:1. Using these substrate amounts allows for achieving the appropriate viscosity of the recovered polyol. It is worth noting that glycolysis of flexible foams resulted in a split-phase glycolysate in over 80% of cases. The upper phase was suitable for further processing, while the lower phase contained undesirable reaction products and was considered waste.

In contrast, glycolysis of rigid polyurethane foams almost always results in a single-phase glycolysate. If there is any mention of a two-phase product in such glycolysis, it usually refers to the remnants of undissolved foam due to an insufficient amount of glycol or catalyst used. The most effective catalysts for decomposing rigid foam have been found to be alkaline metal hydroxides, which were most frequently chosen in experiments. In several cases, the titanium catalyst demonstrated equally high catalytic efficiency. The most commonly used alcoholysis agents in this context were glycols with low molecular weight and high polarity. Emerging trends in polyurethane foam decomposition involve combining several different chemical methods. The most commonly used combined methods include glycolysis and acidolysis. By combining these methods, it is possible to obtain glycolysates of higher purity, characterized by lower acid numbers and lower concentrations of undesirable aromatic amines, which are known to be carcinogenic. It is worth noting that methods to reduce energy consumption during the glycolysis process are also being explored. As a result, replacing traditional heating of the reaction mixture with microwave (MW) irradiation has been tested. This approach significantly reduced the foam dissolution time and accelerated the reaction rate by more than 20 times. Currently, active patents describe many interesting solutions for adapting glycolysate for further production use. These include methods such as combined glycolysis, the creation of a preliminary prepolymer or dispersion in a commercial polyol, or the addition of higher-functionality alcohols during the glycolysis stage to increase the overall functionality of the recovered polyol. The obtained glycolysate is generally used for the production of new rigid polyurethane foams, other insulating materials, elastomers, or protective coatings.

Chemical recycling of foamed polyurethane materials is a critical issue in this era of rapidly advancing technology across various sectors of the economy. Existing and new regulations, particularly under the Green Deal framework, compel manufacturing companies to adopt more environmentally friendly waste management policies. Moreover, companies are competing to develop increasingly modern and sustainable products to maintain a strong market position for their brand or product. In this growing environment, the issue of chemical recycling of PU foams will undoubtedly continue to develop, especially glycolysis, which offers many possibilities due to its flexibility. Implementing recycling through chemical depolymerization enables a circular economy, providing additional value in the form of a new sustainable and competitive product. This review serves as an ideal foundation for starting research in this direction, both on the glycolysis process itself and on the substrate, specifically semi-rigid polyurethane foam, which has been entirely neglected in previous experiments. An important aspect worth investigating is the creation of new high-quality polyurethane materials that ensure 100% utilization of recycled polyols.

## Figures and Tables

**Table 1:** Advantages and disadvantages of the possibilities for PU recycling.

Methods of PU Recycling	Advantages	Disadvantages
Physical methods [54]	- Low cost and simplicity of the process - Does not require advanced technological infrastructure - Minimal energy consumption	- Limited processing capabilities—the resulting material can only be used as an additive or filler - Low quality of the recovered material
Thermochemical methods [56]	- Efficient disposal of large quantities of waste - Potential for energy recovery in the form of heat	- Emission of harmful substances (e.g., dioxins, NOx, CO) - Loss of material—no recovery of original raw materials - High costs associated with flue gas filtration and purification
Hydrolysis [57]	- Ability to obtain basic raw materials that can be reused in production	- Requires high temperatures and pressures, increasing energy costs - The process can be time-consuming. The reaction product is difficult to manage. Low profitability. - Large amounts of carcinogenic amines are formed
Methanolysis [60]	- Ability to obtain basic raw materials that can be reused in production	- The process demands precise control conditions
Aminolysis [61]	- Production of high-quality products such as amines and polyols - Capable of processing different types of PU - Process carried out without an additional catalyst	- Requires the use of toxic amines, posing health and environmental risks - High operational costs
Acidolysis [62]	- Ability to produce products with diverse chemical functionality - The process can be applied to various types of PU	- Involves the use of strong acids, which can be corrosive and difficult to manage
Glycolysis [67]	- High efficiency in recovering high-quality raw materials (polyols) - Applicable to a wide range of substrates and catalysts - Well suited for industrial scale-up - Relatively mild reaction conditions	- Requires the use of catalysts, which can increase process costs - Potential contamination issues with the recovered material

**Table 2:** Overview of flexible foam glycolysis.

Glycol	Catalyst	Temp. °C	PU/ Glycol Ratio	Properties/ Advantages	Split- or Single-Phase	Ref.
diethylene glycol (DEG)	octoate salts: Li, Sn, K, Ca, Sr, Co, Ni and Zn	190	1:1.5	Lithium and stannous octoates demonstrated remarkable catalytic activity and the highest decomposition rates.	2	[78]
GPX-600 (Mw: 600 Da), GPX-3000 (Mw: 3000 Da), and GPX-6000 (Mw: 6000 Da)	potassium hydroxide (KOH)	195	1:1, 1:1.5, 1:2	Regardless of the amount of glycolytic agents used, an increase in catalyst content results in a higher conversion rate. The optimal glycolysis condition was determined to be a catalyst concentration of 1.0 wt.%.	It depends on the glycol used.	[84]
dipropylene glycol (DPG)	KOH	180–210 °C	13:1, 4:1	Propoxylation of received glycolysate.	1	[85]
DEG	diethanolamine (DEA)	170 °C, 190 °C, 210 °C	1:1.25, 1:1.5, 1:2	The best result was with the smallest amount of DEA (DEA/DEG = 1:9) and a temperature of 210 °C.	2	[71]
ethylene glycol (EG), propane 1,3 diol (PDO), butane 1,4 diol (BDO), pentane-1,5-diol, hexane-1,6-diol (HDO)	potassium acetate (KAc)	190–250 °C	10:1	The type of glycol has a minimal impact on the chemical structure of the glycolysate, while the thermal stability increases with the use of glycols with higher molecular weight.	2	[86]
glycerin (GLY) (crude and refined)	dibutyltin dilaurate (DBTDL), stannous octoate (Sn(Oct)2), sodium hydroxide (NaOH), KAc, 1,4-diazabicyclo [2.2. 2]octane (DABCO), triethanolamine (TEA)	220	3:1	The optimal conditions were determined as follows: reaction time of 60 min at 220 °C using dibutyltin dilaurate catalyst (0.5%).	2	[87]
DEG	Sn(Oct)2	179–189	1:0.9, 1:1.125, 1:1.5	The optimal ratio of PU/glycol was 1:1.125 and the temperature was 189 °C.	2	[88]
DEG	Sn(Oct)2	190	1:1.5	The recovered polyol, after purification, exhibited parameters similar to the original raw materials.	2	[89]
GLY	Sn(Oct)2	190	1:1.5	Upper phase with higher purity compared to using DEG as the glycolysis agent	2	[90]
DEG	KAc	215–225	1:0.5, 1:1, 1:1.5, 1:2, 1:2.5	The optimal amount of catalyst was 1% KAc/PU.	2	[91]
GLY	KOH, NAOH	160, 180, 200, 220 °C	1:1	The use of microwaves significantly reduced the glycolysis time, and better results were achieved with NaOH.	2	[92]
DEG	Zn/Sn/Al hydrotalcite (HTC)	180, 185, 190	1.5:1	The highest amount of recovered polyol was achieved for a temperature of 190 °C, a 3 h reaction, and 0.01 g of hydrotalcite.	2	[73]
DEG	DEA	180, 190	1:3	Testing three different methods: atmospheric pressure, autoclave, and high frequency.	1	[93]
monoethylene glycol (MEG), DEG, 1,2-propylene glycol (MPG), and dipropylene glycol isomer mixture	DEA	189	1:1.5	Diethylene glycol was the most effective.	2	[80]
DEG	DEA, titanium(IV) butoxide (TnBT), potassium octoate (KOc), and calcium octoate (Ca(Oc)_2_)	189	1:1.5	Potassium octoate proved to be the most promising catalyst.	2	[94]
DEG	KOc	175-195	1:0.75–1:2	The most effective parameters were catalyst of 2.2%, temperatures in the range of 180–200 °C, and a foam/glycol ratio of 1:1.5.	2	[95]
DEG, EG, pentaerythritol, GLY, diglycerol	Bi(III) neodecanoate	200	1:0.5, 1:1, 1:1.5	Adding lactam to PU foam in a weight ratio of 0.1:1 reduced the dissolution time of PU foam.	2	[83]
crude GLY	Sn(Oct)2	190	1.5:1	Scaling up the process to a pilot scale, a product with a purity of >96% by weight of recovered polyol was obtained after purification.	2	[96]
PG	NaOH	180, 200	1:2, 1:3, 1:4	Recovered polyol obtained at a PG/PU weight ratio of 3:1 at 200 °C showed similar properties to those obtained at a PG/PU weight ratio of 4:1 at 180 °C.	2	[82]
triblock polyether (Triblock Polyoxypropylene Polyoxyethylene Copolymer, PEG-PPG-PEG) L31, polyether polyols (HSH-210)	DBTDL	180	1:1, 1:1.5, 1:2, 1:3, 1:4	The optimum reaction conditions for homogeneous degradation product were investigated as follows: DBTDL 1% mass ratio, L31/PU ratio 1.5:1, 180 °C, and 12 h reaction time.	1	[97]

**Table 3:** Overview of rigid foam glycolysis.

Glycol	Catalyst	Temp. °C	PU/Glycol Ratio	Properties/Advantages	Split- or Single-Phase	Ref.
MPG, tripropylene glycol, EG, DEG, triethylene glycol, tetraethylene glycol, PEG, triethylamine (TEA), DPG, polypropylene glycol (PPG), G400, C, 1,4-butanediol, 1,2-butanediol, HDO, VORANOL 446, VORANOL RN482, VORANOL 391, VORANOL RA640	NaOH, KOH, sodium acetate (NAc), KAc, DBTDL, TnBT, Sn(Oct)2	170–200	1:1, 1:1.3, 1:2	KOH was the most effective catalyst. The best aging.	No information	[100]
EG, DEG	NaAc, NaOH, TEA	240	1:1.5	The optimal glycolysis conditions are a weight ratio of EG/PU 1:1, NaOH/PU = 1%, a temperature of 197.85 °C, and a reaction time of 2 h.	1	[101]
DPG	KAc, TnBT	180–220	1:3, 1:1.5, 1.2:1	KAc proved to be a more suitable catalyst; the optimal PU/glycol ratio was 1:1.5.	1	[102]
EG, DEG	KOH, and titanium glycolate Ti(OCH_2_CH_2_O)_2_	180	1:1	The mass ratio of mixture EG:DEG = 6:4. Titanium glycolate proved to be a more effective catalyst.	1	[103]
EG, MPG, BDO	KOH, TnBT	180	1:1	Using a three-component alcoholic solubilizer ratio EG:PDO:BDO = 35:35:30, the titanium catalyst demonstrated generally higher catalytic efficiency.	1	[104]
EG, MPG	NaOH, duplex metal catalysts (DMCs)	220	1:1	Various mass ratios of EG:MPG mixtures were used: 70:30, 60:40, 50:50, and 40:60.	1	[72]
GLY, BDO	Cesium hydroxide (CsOH), KOH	170	1:1, 1:1.1, 1:1.2, 1:1.3, 1:1.5	The best ratio of GLY:BDO is 3:2 to achieve the best reaction effect.	1	[105]
DEG	KAc, tin(II)2-ethylhexanoate, SnOct2, monoethanolamine (MEA)	200	1:1.5	The optimal glycolysis conditions are a weight ratio of DEG/PU 1:1, NaOH/PU = 1%, a temperature of 197.85 °C, and a reaction time of 2 h.	1	[106]
EG, DEG	NaOH, NaAc, DEA	180, 198	1:4	The most effective parameters were using EG with NaOH for a 2 h reaction time. A solid phase was obtained, which was then filtered and centrifuged.	2	[107]
EG	NaOH	180, 198	1:1.5, 1:4	The recovered polyols had higher hydroxyl numbers, acid numbers, average molecular weights, and viscosities compared to pure synthetic polyols.	1	[108]
GLY, DEG	Ionic liquids (LIMn, LIZn), NaOH, niobium pentoxide (Nb2O5), niobium (V) chloride (NbCl5), and niobium ammoniacal oxalate	195–205	1:5	NaOH proved to be the most effective catalyst. Glycerol was the best option compared to DEG.	1	[109]
DEG	KAc	200	1.5:1	An optimal KAc concentration of 30 mmol/100 g of polyurethane was the best way along with the concentration of methylenedianilne (MDA) and efficient depolymerization degree.	1	[81]

**Table 4:** Overview of active patents in glycolysis.

Publication Number	Current Assignee	Publication Date	The Method Used/Benefits	Ref.
ES2277554B1	Poliuretanos	16 July 2008	Combined glycolysis–acidolysis. The glycolysis products are treated with a blend of organic acids to minimize the presence of unwanted primary aromatic amines in the final product.	[130]
KR101061839B1	Hanbat Nat Univ Ind Academic Cooperation Found	05 September 2011	The use of a short diol as the glycolysis agent, along with the addition of pentaerythritol or sorbitol, increases the functional groups of the recycled polyol and ensures increased compressive strength in the new polyurethane.	[131]
EP2183311B1	Astav Makromolekularna Chem Av Cr V V I	03 December 2014	Using microwave heating and a low-molecular-weight polyhydric alcohol for faster decomposition of PU foam and reducing energy consumption.	[132]
US8609740B2	International Automotive Components Group North America, Inc.	17 December 2013	Adding a viscosity reducer, particularly a dibasic ester, to bring the mixture’s viscosity to the optimal range for subsequent processing.	[133]
CN106977765B	Hunan Zhixin Ester New Material Co., Ltd. (Zhuzhou City, China)	28 August 2018	The addition of an alcoholysis aid (N-methylmorpholine, phosphite, and carbodiimide or butylated diphenylamine, octylated diphenylamine) along with the glycolysis agent ensures the processing of various types of PU materials.	[134]
US11124623B2	H&S Anlagentechnik Gmbh	21 September 2021	Combined acidolysis–glycolysis. First, acidolysis occurs, followed by glycolysis, resulting in a polyol with a low acid number.	[135]
CN113429540B	Zibo Huashiyuan Environmental Protection Tech Co., Ltd. (Zibo, China)	04 October 2022	Using a two-component alcoholysis agent along with an alcoholysis assistant to obtain better thermal insulation properties in new PU materials.	[136]
KR1020230042812A	Jung Woo Fine	30 March 2023	Using an aldehyde compound to react with the glycolysis product to reduce the reactivity resulting from the presence of amines.	[137]
KR1020230041371A	Jung Woo Fine	24 March 2023	Using an isocyanate component to react with the glycolysis product to reduce the reactivity resulting from the presence of amines.	[138]
CN113354863B	Shandong Dongte Environmental Tech Co., Ltd. (Liaocheng, China)	26 May 2023	Using three different alcoholysis agents (from the groups of glycols, polyethers, or polyesters; cyclic alcohols; and polyhydroxy alcohols) along with an alcoholysis aid (amine compounds). The obtained polyol can be used directly to produce new PU material.	[139]
CN115260580B	Zhejiang Univ Of Tech	02 February 2024	Combined glycolysis–acidolysis. Designing a specialized device for the degradation of PU waste that addresses the issues of mass and heat transfer during foam degradation.	[140]

## Data Availability

Not applicable.
